# Outcomes following major thoracoabdominal cancer resection in adults with congenital heart disease

**DOI:** 10.1371/journal.pone.0295767

**Published:** 2024-01-02

**Authors:** Sara Sakowitz, Syed Shahyan Bakhtiyar, Konmal Ali, Saad Mallick, Catherine Williamson, Peyman Benharash

**Affiliations:** 1 Cardiovascular Outcomes Research Laboratories (CORELAB), University of California, Los Angeles, CA, United States of America; 2 Department of Surgery, University of Colorado, Aurora, CO, United States of America; 3 Department of Surgery, University of California, Los Angeles, CA, United States of America; The University of Tokyo Graduate School of Medicine, JAPAN

## Abstract

**Background:**

While advances in medical and surgical management have allowed >97% of congenital heart disease (CHD) patients to reach adulthood, a growing number are presenting with non-cardiovascular malignancies. Indeed, adults with CHD are reported to face a 20% increase in cancer risk, relative to others, and cancer has become the fourth leading cause of death among this population. Surgical resection remains a mainstay in management of thoracoabdominal cancers. However, outcomes following cancer resection among these patients have not been well established. Thus, we sought to characterize clinical and financial outcomes following major cancer resections among adult CHD patients.

**Methods:**

The 2012–2020 National Inpatient Sample was queried for all adults (CHD or non-CHD) undergoing lobectomy, esophagectomy, gastrectomy, pancreatectomy, hepatectomy, or colectomy for cancer. To adjust for intergroup differences in baseline characteristics, entropy balancing was applied to generate balanced patient groups. Multivariable models were constructed to assess outcomes of interest.

**Results:**

Of 905,830 patients undergoing cancer resection, 1,480 (0.2%) had concomitant CHD. The overall prevalence of such patients increased from <0.1% in 2012 to 0.3% in 2012 (P for trend<0.001). Following risk adjustment, CHD was linked with greater in-hospital mortality (AOR 2.00, 95%CI 1.06–3.76), as well as a notable increase in odds of stroke (AOR 8.94, 95%CI 4.54–17.60), but no statistically significant difference in cardiac (AOR 1.33, 95%CI 0.69–2.59) or renal complications (AOR 1.35, 95%CI 0.92–1.97). Further, CHD was associated with a +2.39 day incremental increase in duration of hospitalization (95%CI +1.04–3.74) and a +$11,760 per-patient increase in hospitalization expenditures (95%CI +$4,160–19,360).

**Conclusions:**

While a growing number of patients with CHD are undergoing cancer resection, they demonstrate inferior clinical and financial outcomes, relative to others. Novel screening, risk stratification, and perioperative management guidelines are needed for these patients to provide evidence-based recommendations for this complex and unique cohort.

## Introduction

Advances in the cross-disciplinary management of those with congenital heart disease (CHD) have allowed >97% of such patients to survive into adulthood [1]. As a consequence of improving survival, the incidence of adults CHD patients presenting with non-cardiovascular diseases and malignancy has also increased [2, 3]. In a study of ∼89,000 adults, Karazisi and colleagues [4] found a ∼20% increase in risk of cancer among CHD patients, even after adjusting for genetic syndromes and organ transplantation. In addition to increased exposure to radiation from diagnostic or therapeutic procedures in childhood, prior work has suggested CHD and cancer may share certain genetic or environmental risk factors [5]. Ultimately, an amalgamation of such aspects have led cancer to become the fourth leading cause of death among CHD patients [6, 7].

Upon cancer diagnosis, many patients may be offered surgical resection as part of disease management. While often part of gold standard, multi-modal approaches, these procedures can be associated with >10% risk of serious adverse events, including postoperative bleeding, infection, or embolism [8–10]. Given their unique cardiac architecture, subsequent suboptimal physiologic reserve, and associated hepatopulmonary dysfunction in certain subtypes, patients with CHD may be at heightened risk for death or major complications following such oncological resections [11, 12]. Yet, to date, outcomes of major cancer resections among patients with CHD have not been well established in the literature. Thus, both clinicians and patients remain without a clear understanding of the additional perioperative challenges these patients may face.

In the present study, we examined outcomes following major resection for lung, esophageal, gastric, pancreatic, hepatocellular, and colon cancer using a nationally-representative and contemporary sample. We hypothesized that patients with CHD would face greater in-hospital mortality, perioperative complications, and resource utilization.

## Methods

All records for adult (≥18 years) hospitalizations entailing diagnosis codes for major cancers and associated procedure codes for cancer resections, including lobectomy for lung cancer, esophagectomy for esophageal cancer, gastrectomy for gastric cancer, pancreatectomy for pancreatic cancer, hepatectomy for hepatocellular carcinoma, and colectomy for colon cancer, were identified from the 2012–2020 National Inpatient Sample (NIS) using previously reported *International Classification of Diseases*, *Ninth and Tenth Revision* (ICD-9-CM and ICD-10-CM) diagnosis and procedure codes [13]. As the largest publicly available, all-payer database, the NIS provides accurate estimates for ∼7 million hospitalizations and >97% of the United States population each year [14].

**Fig 1:**
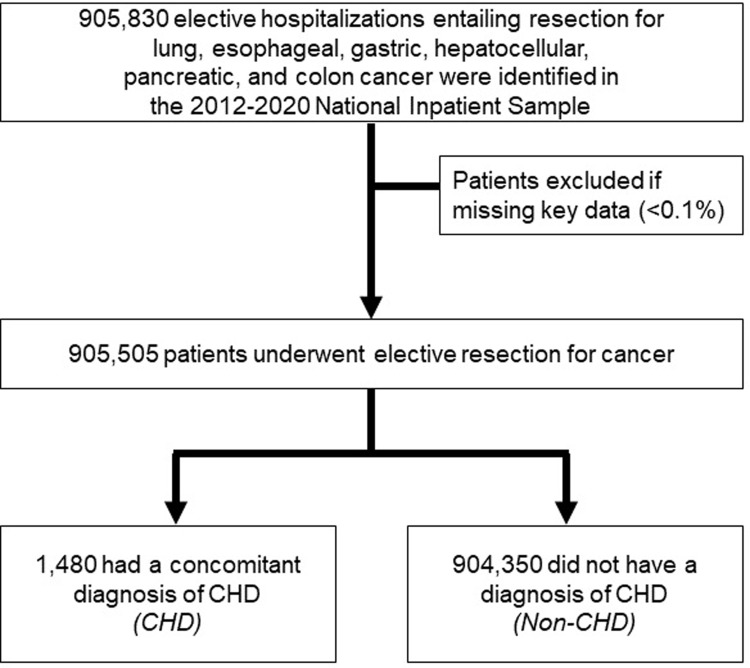
CONSORT diagram of survey-weighted estimates. Of 905,830 hospitalizations for elective resection for lung, esophageal, gastric, hepatocellular, pancreatic, and colon cancer tabulated in the 2012–2020 NIS, 1,480 (0.1%) had a prior congenital heart disease (CHD) diagnosis. All estimates represent survey-weighted methodology.

Individual diagnoses of CHD diagnoses were ascertained using appropriate ICD-9-CM/10-CM codes as previously published [11, 15]. Single-ventricle CHD comprised hypoplastic left heart syndrome, tricuspid atresia, Ebstein’s anomaly, pulmonary valve atresia, common ventricle, and double-outlet right ventricle, except for tetralogy of Fallot and transposition of the great arteries (S1 Table) [16]. Records missing key data were excluded (<0.1%) (Fig 1).

### NIS, National Inpatient Sample

The HCUP data dictionary was utilized to define patient and hospital factors [14]. The well-validated Elixhauser Comorbidity Index was applied to quantify patient burden of chronic conditions at hospitalization [17]. Relevant comorbidities and perioperative complications were tabulated using ICD-9-CM/10-CM codes [13]. Hospital annual cancer resection case volume was calculated and stratified into terciles. Center-specific, cost-to-charge ratios were used to calculate overall hospitalization costs, and subsequently inflation-adjusted using the 2020 Personal Healthcare Price Index [18].

The primary outcome of the study was in-hospital mortality during index admission for resection. Secondary outcomes included the development of perioperative complications (cardiac, renal, stroke, infectious, respiratory, thrombotic, and need for blood transfusion), duration of hospitalization (LOS), and hospitalization costs.

Patient and hospital characteristics were compared using Pearson’s, Mann-Whitney *U*, and adjusted Wald tests. To adjust for baseline variation, entropy balancing was utilized. Briefly, this method applies pseudo-propensity scores to balance patient and hospital factors between groups while retaining the entire cohort for analysis [19]. Of note, entropy balancing has been demonstrated to be more statistically robust than propensity matching [20]. Multivariable regression models were subsequently constructed to assess the independent association of CHD with outcomes of interest. Model covariates were selected using Elastic net regularization, which utilizes a penalized least-squares methodology to reduce model overfitting and covariate collinearity [21]. Variables ultimately selected for inclusion included patient age, sex, Elixhauser comorbidity index, insurance coverage, zipcode-based income quartile, procedure type, year of diagnosis, and the presence of comorbid coronary artery disease, pulmonary circulation disorders, neurological disorders, chronic pulmonary disease, liver disease, and coagulopathic disorders, as well as hospital teaching status. Receiver operating characteristics were used to examine model discrimination. Logistic and linear regression model outputs are presented as adjusted odds ratios (AOR) and beta-coefficients (β), respectively, both with 95% confidence intervals (95%CI). We did not correct for multiplicity, and as such generalizable conclusions cannot be assumed from the width of confidence intervals. All reported estimates represent survey-weighted methodology.

Statistical significance was set at α = 0.05. All statistical analyses were performed using Stata 16.1 (StataCorp, College Station, TX). Due to the fully de-identified nature of the NIS, this study was exempted from full review by the Institutional Review Board at the University of California, Los Angeles.

## Results

**Fig 2:**
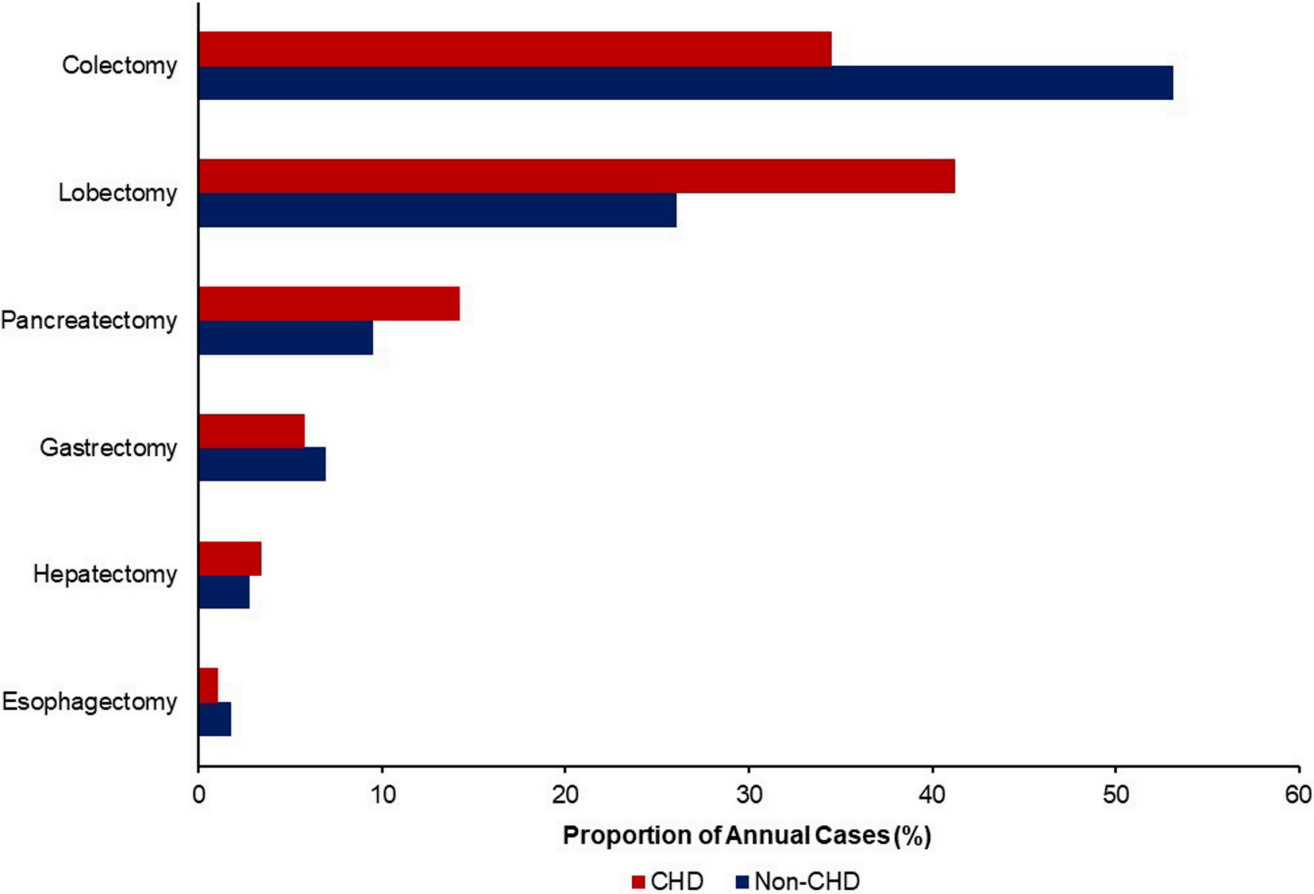
Cancer breakdown by CHD. Relative to non-CHD, patients with CHD more frequently underwent lobectomy (41.2 vs 26.0%), pancreatectomy (14.2 vs 9.5%), and hepatectomy (3.4 vs 2.8%). However, they less often underwent colectomy (34.5 vs 53.1%) and esophagectomy (1.0 vs 1.8%, P<0.001).

Of an estimated 905,830 patients admitted for major cancer resection, 0.2% (1,480) had a diagnosis of CHD. Among CHD patients, 115 (7.8%) had single-ventricle disease. The prevalence of patients with CHD undergoing resection for cancer increased from <0.1% in 2012 to 0.3% in 2019 and 0.3% in 2020 (P for trend<0.001). Compared to non-CHD patients, adult cancer patients with CHD more frequently underwent lobectomy for lung cancer (41.1 vs 26.0%) and pancreatectomy for pancreatic cancer (14.2 vs 9.5%, P<0.001, Fig 2).

### CHD, congenital heart disease

The CHD cohort was, on average, similar in distribution of age, sex, income, and insurance coverage, compared to non-CHD. Relative to non-CHD, patients with CHD presented with a higher Elixhauser comorbidity index (4 [3–5] vs 3 [2–5], P<0.001) and more often faced coronary artery disease (25.0 vs 15.6%, P<0.001), chronic lung disease (28.7 vs 23.3%, P = 0.03), cerebrovascular disease (3.3 vs 6.1%, P = 0.01) and coagulopathic disorders (6.1 vs 3.3%, P = 0.01). While CHD and non-CHD were similarly often treated at high volume institutions (70.9 vs 65.1%, P<0.001), they more frequently received care at metropolitan teaching centers (87.5 vs 75.1%, P<0.001). A complete characterization of the cohort is detailed in Table 1.

**Table 1:** Demographic, clinical, and hospital characteristics.

	*Non-CHD* (n = 904,350)	*CHD* (n = 1,408)	*P-value*
Age (years [IQR])	67 [59–75]	69 [61–76]	0.13
Female (%)	48.2	51.0	0.32
Elixhauser Comorbidity Index (years [IQR])	3 [2–5]	4 [3–5]	<0.001
*Cancer resection (%)*			<0.001
Lobectomy	26.0	41.1	
Esophagectomy	1.8	1.0	
Gastrectomy	6.9	5.7	
Colectomy	53.1	34.4	
Pancreatectomy	9.5	14.2	
Hepatectomy	2.8	3.4	
*Income quartile (%)*			0.10
>75%	24.1	30.2	
51–75%	25.3	24.7	
26–50%	26.3	23.3	
0–25%	24.4	21.9	
*Insurance coverage (%)*			0.33
Private	33.0	28.0	
Medicare	57.2	61.8	
Medicaid	6.2	6.8	
Other Payer	3.5	3.4	
*Comorbidities (%)*			
Coronary artery disease	15.6	25.0	<0.001
Cardiac arrhythmias			
Pulmonary circulation disorders	1.8	7.8	<0.001
Cerebrovascular disease	3.3	6.1	0.01
Chronic lung disease	23.3	28.7	0.03
Diabetes	22.7	18.9	0.12
Anemia	6.2	5.4	0.56
Liver disease	5.2	4.4	0.52
Coagulopathies	3.3	6.1	0.01
*Hospital teaching status (%)*			<0.001
Urban teaching	75.1	87.5	
Urban non-teaching	18.6	8.4	
Rural	6.3	4.1	

Reported as proportions unless otherwise noted. Statistical significance was set at α = 0.05.

*SD, standard deviation; *IQR*, inter-quartile range

Following resection, CHD patients more often experienced in-hospital mortality (3.4 vs 1.3%, P = 0.002), as well as stroke (3.0 vs 0.3%, P<0.001), and renal complications (10.5 vs 6.5%, P<0.001), compared to their non-CHD counterparts. Additionally, CHD patients demonstrated greater median LOS (6 [4–10.5] vs 5 [4–8], P<0.001) and costs ($27,300 [$19,700–43,800] vs $20,500 [$14,300–30,800], P<0.001).

**Fig 3:**
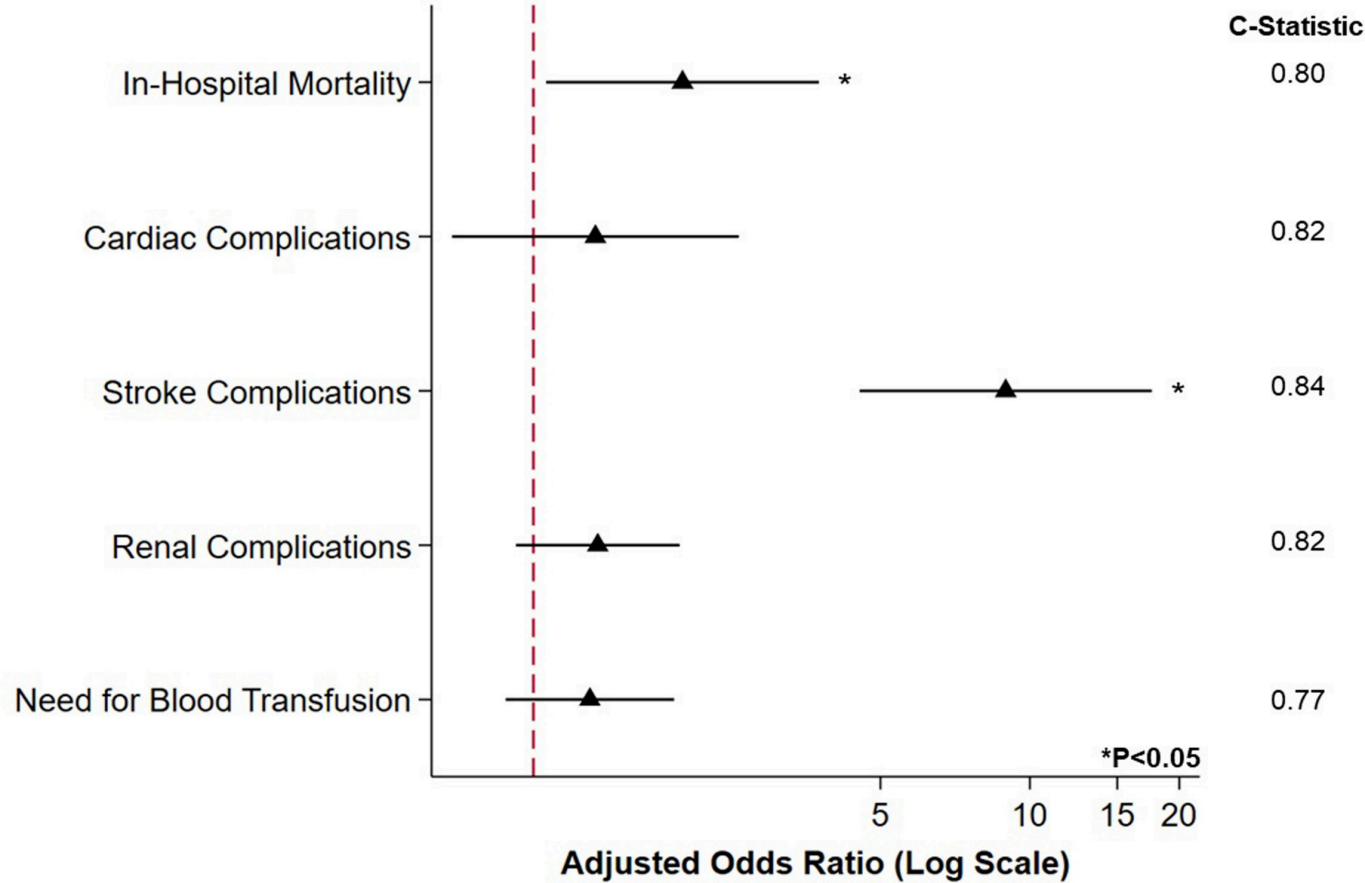
Association of CHD with inferior clinical outcomes. After entropy balance and risk adjustment, prior congenital heart disease (CHD) diagnosis was linked with greater likelihood of in-hospital mortality and stroke. No difference in odds of cardiac or renal complications was observed. * indicates statistical significance, P<0.05. Reference: Non-CHD. Error bars represent 95% confidence intervals.

After entropy balancing, adequate covariate balance was achieved. Following risk adjustment, CHD demonstrated increased odds of in-hospital mortality (AOR 2.00, 95%CI 1.06–3.76; Model C-Statistic 0.80) as well as greater likelihood of stroke (AOR 8.94, 95%CI 4.64–17.60; Model C-Statistic 0.84) (Fig 3).

**Fig 4:**
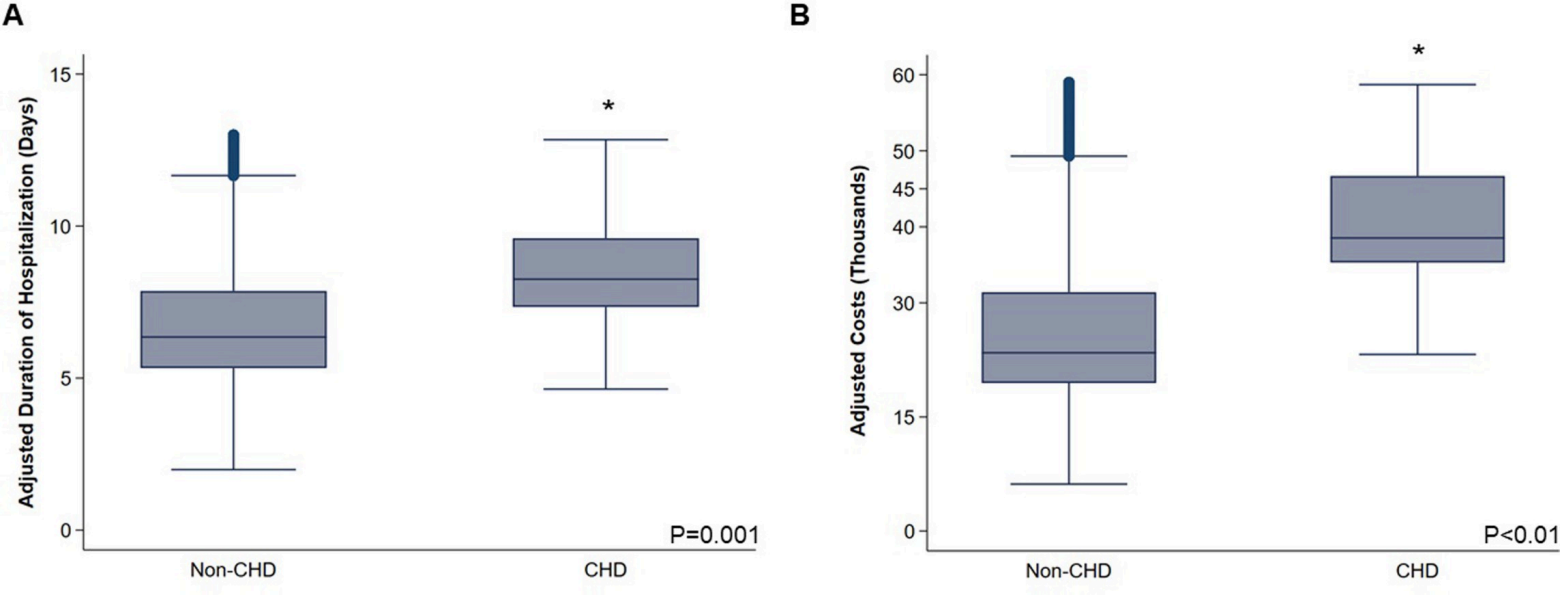
CHD cohort linked with greater resource utilization. After adjustment, the cohort of patients with concomitant congenital heart disease (CHD) demonstrated (A) a +2.39 day incremental increase in duration of hospitalization (95%CI +1.04–3.74), as well as (B) a +$11,760 per-patient increase in hospitalization expenditures (95%CI +$4,160–19,360).

Considering resource utilization, CHD was associated with longer duration of hospitalization (+2.39 days, 95%CI +1.04–3.74) and greater per-patient expenditures (β+$11,760, 95%CI +$4,160–19,360) (Fig 4 and Table 2).

**Table 2:** Unadjusted and adjusted outcomes of the CHD cohort as compared to Non-CHD.

	Unadjusted			Adjusted		
	*Non-CHD*	*CHD*	*P*	*CHD*	*95% CI*	*P*
**Clinical outcomes**						
In-hospital mortality	1.3	3.4	0.002	2.00	1.06–3.76	0.03
Cardiac complications	1.8	3.0	0.09	1.33	0.69–2.59	0.40
Stroke complications	0.3	3.0	<0.001	8.94	4.54–17.60	<0.001
Renal complications	6.5	10.5	0.006	1.35	0.92–1.97	0.12
Infectious complications	3.6	4.4	0.46	1.30	0.74–2.27	0.36
Respiratory complications	9.1	10.1	0.53	0.97	0.66–1.42	0.87
Blood transfusion	8.7	9.5	0.63	1.30	0.88–1.92	0.19
Thrombotic complications	0.9	2.0	0.03	0.81	0.36–1.82	0.61
Non-home discharge	10.0	10.5	0.78	0.94	0.64–1.39	0.77
**Resource utilization**						
Length of stay (days) [IQR]	5 [4–8]	6 [4–10.5]	<0.001	+2.39	1.04–3.74	0.001
Cost (USD $1,000) [IQR]	20.5 [14.3–30.8]	27.3 [19.7–43.8]	<0.001	+11.76	4.16–19.36	0.002

Outcomes reported as proportions or as Adjusted Odds Ratio (AOR) with 95% confidence intervals (95% CI).

*IQR, interquartile range; *USD*, United States dollar

## Discussion

Due to significant advances in medical and surgical management, the last several decades have seen patients with CHD experience a dramatic improvement in survival [2, 22]. Yet, these patients are noted to face both an increased non-cardiac disease burden, as well as greater perioperative risk, relative to others [2, 7, 15, 23]. Thus, the present study characterized clinical and financial outcomes of major cancer resections in a contemporary national sample of adults with CHD. We report these patients faced higher in-hospital mortality and perioperative complications. CHD was further linked with a +2.56 day increase in duration of hospitalization and a ∼$16,000 increase in per-patient expenditures. Several of these findings merit further discussion.

Prior work has established the increased risk of cancer in patients with CHD relative to the general population [4, 22–27]. Indeed, in a study of 31,961 patients in Taiwan, CHD patients demonstrated a 45% increase in relative risk of malignancy over five years of follow-up [28]. Similarly, Mandalenakis et al. report a doubling in the risk of developing a malignancy among CHD patients followed for nearly 40 years [24]. Potentially reflecting this literature, we identified an increasing trend in the number of CHD patients undergoing cancer resection from 2010 to 2020. Considering recent studies have suggested patients undergoing reparative interventions in infancy may face even greater low-dose radiation burden with unknown long-term sequelae [22, 29], we postulate the incidence of cancer may grow higher among newer generations of CHD patients [30]. Future studies should investigate differences in cancer risk relative to timing of surgical procedures in this cohort.

Additionally, we report resection for lung cancer was most prevalent among the CHD cohort, in accordance with prior reports [22, 24, 31]. These patients, in particular those palliated with the Fontan procedure, may face low cardiac output and higher systemic venous pressures, potentially enhancing carcinoma development [32, 33]. Notably, however, there is a significant gap in relevant guideline-based screening for these patients [34]. In a single-center study, only 16% of CHD patients undergoing cancer treatment had undergone prior screening [35]. Specific screening protocols for adult CHD patients should reflect the multiple risk factors and the increased radiation exposure that patients with CHD endure [34, 36].

Further, after adjusting for relevant patient and hospital factors, we found CHD patients to demonstrate greater in-hospital mortality and perioperative complications following cancer resection, relative to non-CHD. These findings align with previous work that has demonstrated CHD patients to demonstrate greater operative risk for morbidity following a number of elective procedures for various indications [15, 37]. Indeed, at baseline, patients with CHD present with worse physical and cardiovascular functional status that may make them more susceptible to complications [32, 33]. While increased risk may originate from certain unmodifiable factors that engender decreased perfusion, such as worse ventricular function or arrhythmias, avoiding the need for bypass may reduce risk among these patients [38]. We also identified a notable increase in likelihood of postoperative stroke among the CHD cohort, in concordance with literature reporting adult CHD patients face greater propensity for cerebrovascular disease, relative to the general population [39]. This increased risk has been suggested to stem from right-to-left shunts, prior palliative procedures, or the erythrocytosis secondary to cyanotic disease [40]. Intraoperatively, these patients may experience increases in pulmonary vascular resistance, shunting, right ventricular dysfunction, or inadequate cardiac output, potentially leading to embolism, decreased perfusion, and multi-organ complications [41]. Yet, there is a significant lack of evidence regarding optimal risk-assessment, anesthetic management, or hemodynamic monitoring of these patients [37]. Given the impact of hypoxemia, hypercarbia, and volume shifts for these patients, in particular for those with complex or univentricular lesions, novel approaches are needed to improve pre-, intra-, and post-operative care.

Broadly, patients with CHD demonstrate higher rates of healthcare utilization both in the inpatient and outpatient contexts [42, 43]. In the present study, we report an approximately $11,760 increase in hospitalization expenditures for each CHD patient, even after adjusting for the development of perioperative complications. While we did note a ∼2 day incremental increase in duration of hospitalization, we proffer the difference in cost may largely stem from more intensive medical management. Indeed, adults with CHD may face a number of hemodynamic, electrophysiologic, thromboembolic, or neurologic abnormalities stemming from residua or sequelae of corrective surgery that ultimately require multimodal and multi-disciplinary management [42]. We further identified that adjusted per-patient costs for the CHD cohort increased over the study period, from $24,160 in 2012 to over $30,326 in 2020. With a growing number of CHD patients reaching adulthood, most of whom are covered by public insurance, this cohort will therefore comprise an increasing proportion of national hospital expenditures [44]. Thus, novel strategies are needed to maximize quality of care while reducing both hospital and patient financial burden.

The present work has certain limitations inherent to its retrospective nature. The National Inpatient Sample is an administrative database that does not report ejection fraction, echocardiogram findings, or genetic testing. Cancer staging data was unavailable, as was information regarding tumor size, nodal disease, or metastasis. While we could access CHD diagnosis, we could not identify if patients had a history of Fontan or other palliative procedures. Unfortunately, this is a limitation faced by all large-database analyses of CHD due to lack of granular *International Classification of Diseases* codes for history of these procedures. Further, due to insufficient discriminatory detail, *ICD* codes may be unable to classify specific CHD subtypes. While we adjusted our models for annual center volume, we could not account for cumulative center or surgeon experience. Additionally, while we considered a national sample, the small size of the CHD cohort may have contributed to greater variation and imprecision. Yet, despite these caveats, we applied robust and thorough statistical methodology to conduct a large-scale and nationally representative analysis of CHD patients undergoing resection for cancer.

## Conclusion

In conclusion, we report a growing number of patients with CHD undergoing resection for cancer. Patients with CHD demonstrated inferior clinical and financial outcomes following cancer surgery, relative to others. Our work underscores the need for novel approaches to perioperative and intraoperative management that consider these patients’ unique anatomy and physiology. Lastly, new risk stratification guidelines are required to provide evidence-based guidelines for optimal cancer care and allow for informed, comprehensive shared-decision making among this complex, unique, and heterogeneous cohort.

## Supporting information

S1 Table ICD-9/10-CM codes. (DOCX)

## References

[1] Mandalenakis Z, Giang KW, Eriksson P, et al. Survival in children with congenital heart disease: Have we reached a peak at 97%? J Am Heart Assoc. 2020;9(22). doi: 10.1161/JAHA.120.017704 33153356 PMC7763707

[2] Khairy P, Ionescu-Ittu R, MacKie AS, Abrahamowicz M, Pilote L, Marelli AJ. Changing mortality in congenital heart disease. J Am Coll Cardiol. 2010;56(14):1149–1157. doi: 10.1016/j.jacc.2010.03.085 20863956

[3] Afilalo J, Therrien J, Pilote L, Ionescu-Ittu R, Martucci G, Marelli AJ. Geriatric congenital heart disease: Burden of disease and predictors of mortality. J Am Coll Cardiol. 2011;58(14):1509–1515. doi: 10.1016/j.jacc.2011.06.041 21939837

[4] Karazisi C, Dellborg M, Mellgren K, et al. Risk of cancer in young and older patients with congenital heart disease and the excess risk of cancer by syndromes, organ transplantation and cardiac surgery: Swedish health registry study (1930−2017). Published online 2022. doi: 10.1016/j.lanepe.2022.100407 35663362 PMC9156800

[5] Campolo J, Annoni G, Giaccardi M, Andreassi MG. Congenital Heart Disease and the Risk of Cancer: An Update on the Genetic Etiology, Radiation Exposure Damage, and Future Research Strategies. J Cardiovasc Dev Dis. 2022;9(8). doi: 10.3390/jcdd9080245 36005409 PMC9409914

[6] Diller GP, Kempny A, Alonso-Gonzalez R, et al. Survival Prospects and Circumstances of Death in Contemporary Adult Congenital Heart Disease Patients under Follow-Up at a Large Tertiary Centre. Circulation. 2015;132(22):2118–2125. doi: 10.1161/CIRCULATIONAHA.115.017202 26369353

[7] Lui GK, Saidi A, Bhatt AB, et al. Diagnosis and Management of Noncardiac Complications in Adults with Congenital Heart Disease: A Scientific Statement from the American Heart Association. Vol 136.; 2017. doi: 10.1161/CIR.0000000000000535 28993401

[8] Law WL, Choi HK, Lee YM, Ho JWC. The impact of postoperative complications on long-term outcomes following curative resection for colorectal cancer. Ann Surg Oncol. 2007;14(9):2559–2566. doi: 10.1245/s10434-007-9434-4 17522945

[9] Viklund P, Lindblad M, Lu M, Ye W, Johansson J, Lagergren J. Risk factors for complications after esophageal cancer resection: A prospective population-based study in Sweden. Ann Surg. 2006;243(2):204–211. doi: 10.1097/01.sla.0000197698.17794.eb 16432353 PMC1448902

[10] Al-Refaie WB, Parsons HM, Henderson WG, et al. Major cancer surgery in the elderly: Results from the american college of surgeons national surgical quality improvement program. Ann Surg. 2010;251(2):311–318. doi: 10.1097/SLA.0b013e3181b6b04c 19838107

[11] Burstein DS, Shamszad P, Dai D, et al. Significant mortality, morbidity and resource utilization associated with advanced heart failure in congenital heart disease in children and young adults. Am Heart J. 2019;209:9–19. doi: 10.1016/j.ahj.2018.11.010 30639612

[12] Goldberg DJ, Shaddy RE, Ravishankar C, Rychik J. The failing Fontan: Etiology, diagnosis and management. Expert Rev Cardiovasc Ther. 2011;9(6):785–793. doi: 10.1586/erc.11.75 21714609

[13] Pan CS, Sanaiha Y, Hadaya J, Lee C, Tran Z, Benharash P. Venous thromboembolism in cancer surgery: A report from the nationwide readmissions database. Surg Open Sci. 2022;9:58–63. doi: 10.1016/j.sopen.2022.04.005 35669894 PMC9166654

[14] Healthcare Cost and Utilization Project. Overview of the National (Nationwide) Inpatient Sample (NIS). Accessed December 15, 2021. https://www.hcup-us.ahrq.gov/nisoverview.jsp

[15] Williamson CG, Ebrahimian S, Ascandar N, et al. Major elective non-cardiac operations in adults with congenital heart disease. Heart. 2023;109(3):202–207. doi: 10.1136/heartjnl-2022-321512 36175113

[16] Bakhtiyar SS, Sakowitz S, Ali K, et al. Survival after cardiac transplantation in adults with single-ventricle congenital heart disease. JACC. Published online 2023. 10.1016/j.jacc.2023.06.037 37704313

[17] Elixhauser A, Steiner C, Harris DR, Coffey RM. Comorbidity measures for use with administrative data. Med Care. 1998;36(1):8–27. doi: 10.1097/00005650-199801000-00004 9431328

[18] Agency for Healthcare Research and Quality. Using Appropriate Price Indices for Expenditure Comparisons. Accessed March 15, 2022. https://meps.ahrq.gov/about_meps/Price_Index.shtml

[19] Hainmueller J. Entropy Balancing for Causal Effects: A Multivariate Reweighting Method to Produce Balanced Samples in Observational Studies. Polit Anal. 2012;20(1):25–46. doi: 10.1093/pan/mpr025

[20] Zhao Q, Percival D. Entropy Balancing is Doubly Robust: J Causal Inference. 2017;5(1). doi: 10.1515/jci-2016-0010

[21] Zou H, Hastie T. Regularization and variable selection via the elastic net. J R Stat Soc Ser B Stat Methodol. 2005;67(2):301–320. doi: 10.1111/j.1467-9868.2005.00503.x

[22] Cohen S, Gurvitz MZ, Beauséjour-Ladouceur V, Lawler PR, Therrien J, Marelli AJ. Cancer Risk in Congenital Heart Disease-What Is the Evidence? Can J Cardiol. 2019;35(12):1750–1761. doi: 10.1016/j.cjca.2019.09.023 31813507

[23] Raissadati A, Nieminen H, Haukka J, Sairanen H, Jokinen E. Late Causes of Death After Pediatric Cardiac Surgery: A 60-Year Population-Based Study. J Am Coll Cardiol. 2016;68(5):487–498. doi: 10.1016/j.jacc.2016.05.038 27470457

[24] Mandalenakis Z, Karazisi C, Skoglund K, et al. Risk of Cancer among Children and Young Adults with Congenital Heart Disease Compared with Healthy Controls. JAMA Netw Open. 2019;2(7):1–9. doi: 10.1001/jamanetworkopen.2019.6762 31276179 PMC12578487

[25] Olsen M, Garne E, Sværke C, et al. Cancer risk among patients with congenital heart defects: A nationwide follow-up study. Cardiol Young. 2014;24(1):40–46. doi: 10.1017/S1047951112002144 23328503

[26] Bjørge T, Cnattingius S, Lie RT, Tretli S, Engeland A. Cancer risk in children with birth defects and in their families: A population based cohort study of 5.2 million children from Norway and Sweden. Cancer Epidemiol Biomarkers Prev. 2008;17(3):500–506. doi: 10.1158/1055-9965.EPI-07-2630 18296646

[27] Engels EA, Pfeiffer RM, Fraumeni JF, et al. Spectrum of cancer risk among US solid organ transplant recipients. Jama. 2011;306(17):1891–1901. doi: 10.1001/jama.2011.1592 22045767 PMC3310893

[28] Lee YS, Chen YT, Jeng MJ, et al. The risk of cancer in patients with congenital heart disease: A nationwide population-based cohort study in Taiwan. PLoS ONE. 2015;10(2):1–13. doi: 10.1371/journal.pone.0116844 25706872 PMC4338195

[29] Marelli A. Trajectories of care in congenital heart disease ‐ the long arm of disease in the womb. J Intern Med. 2020;288(4):390–399. doi: 10.1111/joim.13048 32323405

[30] Tutarel O, Kempny A, Alonso-Gonzalez R, et al. Congenital heart disease beyond the age of 60: Emergence of a new population with high resource utilization, high morbidity, and high mortality. Eur Heart J. 2014;35(11):725–732. doi: 10.1093/eurheartj/eht257 23882067

[31] Gurvitz M, Ionescu-Ittu R, Guo L, et al. Prevalence of Cancer in Adults With Congenital Heart Disease Compared With the General Population. Am J Cardiol. 2016;118(11):1742–1750. doi: 10.1016/j.amjcard.2016.08.057 27702435

[32] Moons P, Luyckx K, Thomet C, et al. Physical Functioning, Mental Health, and Quality of Life in Different Congenital Heart Defects: Comparative Analysis in 3538 Patients From 15 Countries. Can J Cardiol. 2021;37(2):215–223. doi: 10.1016/j.cjca.2020.03.044 32739453

[33] Ladak LA, Hasan BS, Gullick J, Gallagher R. Health-related quality of life in congenital heart disease surgery in children and young adults: A systematic review and meta-Analysis. Arch Dis Child. 2019;104(4):340–347. doi: 10.1136/archdischild-2017-313653 29572215

[34] Christman MP, Castro-Zarraga M, DeFaria Yeh D, Liberthson RR, Bhatt AB. Adequacy of Cancer Screening in Adult Women with Congenital Heart Disease. ISRN Cardiol. 2013;2013:1–6. doi: 10.1155/2013/827696 23984096 PMC3747419

[35] Venkatesh P, Yan KL, Bravo-Jaimes K, Yang EH, Lluri G. Outcomes of malignancy in adults with congenital heart disease: a single center experience. Cardio-Oncol. 2022;8(1). doi: 10.1186/s40959-022-00144-z 36419184 PMC9685873

[36] Villani A, Greer MLC, Kalish JM, et al. Recommendations for cancer surveillance in individuals with RASopathies and other rare genetic conditions with increased cancer risk. Clin Cancer Res. 2017;23(12):e83–e90. doi: 10.1158/1078-0432.CCR-17-0631 28620009

[37] Maxwell BG, Wong JK, Kin C, Lobato RL. Perioperative outcomes of major noncardiac surgery in adults with congenital heart disease. Anesthesiology. 2013;119(4):762–769. doi: 10.1097/ALN.0b013e3182a56de3 23907357

[38] Kwiatkowski DM, Price E, Axelrod DM, et al. Incidence, risk factors, and outcomes of acute kidney injury in adults undergoing surgery for congenital heart disease. Cardiol Young. 2017;27(6):1068–1075. doi: 10.1017/S1047951116002067 27869053

[39] Lanz J, Brophy JM, Therrien J, Kaouache M, Guo L, Marelli AJ. Stroke in Adults With Congenital Heart Disease: Incidence, Cumulative Risk, and Predictors. Circulation. 2015;132(25):2385–2394. doi: 10.1161/CIRCULATIONAHA.115.011241 26597113

[40] Perloff JK, Marelli AJ, Miner PD. Risk of stroke in adults with cyanotic congenital heart disease. 10.1161/01.cir.87.6.1954 8504509

[41] Cannesson M, Earing MG, Collange V, Kersten JR. Anesthesia for Noncardiac Surgery in Children With Congenital Heart Disease. Anesthesiology2. 2009;111:432–440. doi: 10.1016/B978-0-323-42974-0.00023-9 19602959

[42] Mackie AS, Pilote L, Ionescu-Ittu R, Rahme E, Marelli AJ. Health Care Resource Utilization in Adults With Congenital Heart Disease. Am J Cardiol. 2007;99(6):839–843. doi: 10.1016/j.amjcard.2006.10.054 17350378

[43] Willems R, Werbrouck A, De Backer J, Annemans L. Real-world healthcare utilization in adult congenital heart disease: A systematic review of trends and ratios. Cardiol Young. 2019;29(5):553–563. doi: 10.1017/S1047951119000441 31046858

[44] Opotowsky AR, Siddiqi OK, Webb GD. Trends in Hospitalizations for Adults With Congenital Heart Disease in the U.S. J Am Coll Cardiol. 2009;54(5):460–467. doi: 10.1016/j.jacc.2009.04.037 19628123

