# Supplementary material for: Outcomes following major thoracoabdominal cancer resection in adults with congenital heart disease
Source: PLoS One. 2024 Jan 2;19(1):e0295767. doi: 10.1371/journal.pone.0295767 (PMC10760660; doi:10.1371/journal.pone.0295767)
Supplement: S1 Table — (DOCX) [file pone.0295767.s001.docx]

**S1 TABLE: Administrative *International Classification of Diseases, 9^th^ and 10^th^ Revision* (ICD-9/10-CM) Diagnosis Codes for Congenital Heart Disease**

| ***Diagnosis or Procedure*** | ***ICD-9-CM and ICD-10-CM Codes*** |
| --- | --- |
| ***Cancer Diagnoses and Procedures*** | |
| Lobectomy | 32.4, 32.41, 32.49, 0BTC0ZZ, 0BTD0ZZ, 0BTF0ZZ, 0BTG0ZZ, 0BTH0ZZ, 0BTJ0ZZ, 0BTC4ZZ, 0BTD4ZZ, 0BTF4ZZ, 0BTG4ZZ, 0BTH4ZZ, 0BTJ4ZZ |
| Esophagectomy | 42.4, 42.5, 0DB50ZZ, 0DB54ZZ, 0DB10ZZ, 0DB14ZZ, 0DB20ZZ, 0DB24ZZ, 0DB30ZZ, 0DB34ZZ, 0DT50ZZ, 0DT54ZZ, 0DT10ZZ, 0DT14ZZ, 0DT20ZZ, 0DT24ZZ, 0DT30ZZ, 0DT34ZZ, 0DR507Z, 0DR547Z, 0DXE0Z5, 0DXE4Z5, 0DX80Z5, 0DX84Z5 |
| Colectomy | 17.32, 17.33, 17.34, 17.35, 17.36, 45.73, 45.74, 45.75, 45.76, 45.81, 45.82, 45.83, 48.50, 48.51, 48.52, 48.53, 0DTF4ZZ, 0DTH4ZZ, 0DTK4ZZ, 0DBF4ZZ, 0DBH4ZZ, 0DBK4ZZ, 0DTF0ZZ, 0DTH0ZZ, 0DTK0ZZ, 0DBF0ZZ, 0DBH0ZZ, 0DBK0ZZ, 0DTL4ZZ, 0DBL4ZZ, 0DTL0ZZ, 0DBL0ZZ, 0DTM4ZZ, 0DTG4ZZ, 0DBM4ZZ, 0DBG4ZZ, 0DTM0ZZ, 0DTG0ZZ, 0DBM0ZZ, 0DBG0ZZ, 0DTN4ZZ, 0DBN4ZZ, 0DTN0ZZ, 0DBN0ZZ,  0DTE4ZZ, 0DBE4ZZ, 0DTE0ZZ, 0DBE0ZZ |
| Gastrectomy | 43.5, 43.6, 43.7, 43.9, 0DB40ZZ, 0DB43ZZ, 0DB44ZZ, 0DB47ZZ, 0DB48ZZ, 0DT40ZZ, 0DT44ZZ, 0DT47ZZ, 0DT48ZZ , 0DB60ZZ , 0DB63ZZ, 0DB64ZZ, 0DB67ZZ, 0DB68ZZ, 0DT60ZZ, 0DT64ZZ, 0DT67ZZ, 0DT68ZZ, 0DT70ZZ, 0DT74ZZ, 0DT77ZZ, 0DT78ZZ, 0DB70ZZ, 0DB73ZZ, 0DB74ZZ, 0DB77ZZ, 0DB78ZZ |
| Hepatectomy | 50.22, 50.3, 0FB00ZZ, 0FB03ZZ, 0FB04ZZ, 0FB20ZZ, 0FB23ZZ, 0FB24ZZ, 0FB10ZZ, 0FB13ZZ, 0FB14ZZ, 0FT10ZZ, 0FT14ZZ, 0FT20ZZ, 0FT24ZZ |
| Pancreatectomy | 52.51, 52.52, 52.53, 52.59, 52.6, 52.7, 0FBG0ZZ, 0FBG3ZZ, 0FBG4ZZ, 0FTG0ZZ, 0FTG4ZZ |
| Lung Cancer | 162, 209.21, 231.2, C34, C7A.090, D02.20, D02.21, D02.22 |
| Esophageal Cancer | 150, 230.1, V10.03, C15, D00.1, Z85.01 |
| Colon Cancer | 153, 154, 230.3, 230.4, C18, C19, D01.0, D01.1, C7A.021, C7A.022, C7A.023, C7A.024, C7A.025, C7A.029, C7A.096, C7A.1, C7A.8 |
| Gastric Cancer | 151, 230.2, 209.23, 209.25, V10.04, C16, D00.2, Z85.02, C7A.092, C7A.094 |
| Hepatocellular Carcinoma | 155, 209.26, C22, C7A.095 |
| Pancreatic Cancer | 152, 156, 157, 209.25, 209.30, C24, C25, C7A.1, C7A.8, C17, C7A.094 |
| ***Congenital Heart Disease*** | |
| Coarctation of Aorta | 74710, Q251 |
| Common Ventricle | 7453, Q204 |
| Complete Transposition of Great Vessels | 74510, 74519, Q203, Q208 |
| Congenital Anomalies of the Great Veins | 74740, Q269, Q260, Q261, Q268 |
| Congenital Anomalies of Pulmonary Artery | 7473, Q2571, Q255, Q256, Q258, Q259, Q2579 |
| Congenital Mitral Insufficiency | 7466, Q233 |
| Congenital Mitral Stenosis | 7465, Q232 |
| Congenital Pulmonary Valve Anomaly | 7460, 74609, Q223, Q222 |
| Cor Biloculare | 74570, Q211 |
| Cor Triatriatum | 74682, Q242 |
| Coronary Artery Anomaly | 74685, Q245 |
| Corrected Transposition of Great Vessels | 74512, Q205 |
| Double Outlet Right Ventricle *(except Tetralogy of Fallot and Transposition of the Great Arteries)* | 74511, Q201 |
| Ebstein’s Anomaly | 7462, Q225 |
| Endocardial Defect | 74560, 74569, Q212 |
| Hypoplastic Left Heart Syndrome | 74670, Q234 |
| Interruption of Aortic Arch | 74711, Q2521 |
| Malposition of Heart | 74687, Q240, Q241 |
| Obstructive Anomalies of Heart | 74684, Q248 |
| Ostium Primum Defect | 74561, Q212 |
| Other Bulbus Cordis Anomalies | 74580, Q208¸ Q214 |
| Other Congenital Anomalies | 74689, 74690, Q238, Q248, Q239, Q209, Q249 |
| Other Congenital Anomalies of the Aorta | 74720, 74721, 74729, Q254, Q2541, Q2542, Q2543, Q2544, Q2545, Q2546, Q2547, Q2548, Q2549 |
| Partial Anomalous Pulmonary Venous Connection | 74742, Q263, Q264 |
| Patent Ductus Arteriosus | 7470, Q250 |
| Pulmonary Valve Atresia | 74601, Q220 |
| Pulmonary Valve Stenosis | 74602, 74683, Q221, Q243 |
| Subaortic Stenosis | 74681, Q244 |
| Total Anomalous Pulmonary Venous Connection | 74741, Q262 |
| Tetralogy of Fallot | 7452, Q213 |
| Tricuspid Atresia | 7461, Q229, Q224, Q228 |
| Unspecified Defect of Septal Closure | 74590, Q219 |
| Ventricular Septal Defect | 7454, Q210, I2783 |
| ***Comorbidities*** | |
| Congestive heart failure | 39891, 40201, 40211, 40291, 40401, 40403, 40411, 40413, 40491, 40493, 4254, 4255, 4257, 4258, 4259, 428, I43, I50, I099, I110, I130, I132, I255, I420, I425, I426, I427, I428, I429, P290 |
| Peripheral vascular disease | 0930, 4373, 4431, 4432, 4438, 4439, 4471, 5571, 5579, V434, 440, 441, I70, I71, I731, I738, I739, I771, I790, I792, K551, K558, K559, Z958, Z959 |
| Pulmonary circulation disorders | 416, 4150, 4151, 4170, 4178, 4179, I26, I27, I280, I288, I289 |
| Valvular heart disease | 0932, 7463, 7464, 7465, 7466, V422, V433, 394, 395, 396, 397, 424, A520, I091, I098, Q230, Q231, Q232, Q233, Z952, Z953, Z954, I05, I06, I07, I08, I34, I35, I36, I37, I38, I39 |
| Chronic pulmonary disease | 4168, 4169, 5064, 5081, 5088, 490, 491, 492, 493, 494, 495, 496, 500, 501, 502, 503, 504, 505, I278, I279, J684, J701, J703, J40, J41, J42, J43, J44, J45, J46, J47, J60, J61, J62, J63, J64, J65, J66, J67 |
| Diabetes | 2500, 2501, 2502, 2503, E100, E101, E109, E110, E111, E119, E120, E121, E129, E130, E131, E139, E140, E141, E149, 2504, 2505, 2506, 2507, 2508, 2509, E102, E103, E104, E105, E106, E107, E108, E112, E113, E114, E115, E116, E117, E118, E122, E123, E124, E125, E126, E127, E128, E132, E133, E134, E135, E136, E137, E138, E142, E143, E144, E145, E146, E147, E148 |
| Late-stage kidney disease | V451, Z992, Z9115, 40301, 40311, 40391, 40402, 40403, 40412, 40413, 40492, 40493, 5880, V420, 5855, 5856, 5865, 5866, Z49, N19, I120, N250, Z940, N185, N186, I1311 |
| Liver disease | 07022, 07023, 07032, 07033, 07044, 07054, 0706, 0709, 4560, 4561, 4562, 5722, 5723, 5724, 5728, 5733, 5734, 5738, 5739, V427, 570, 571, I864, I982, K711, K713, K714, K715, K717, K760, K762, K763, K764, K765, K766, K767, K768, K769, Z944, K70, K72, K73, K74, B18, I85 |
| Coagulopathic disorders | 286, 2871, 2873, 2874, 2875, D65, D66, D67, D68, D691, D693, D694, D695, D696 |
| Cerebrovascular disorders | 33392, 3319, 3320, 3321, 3334, 3335, 3481, 3483, 7803, 7843, 3362, 334, 335, 340, 341, 345, G254, G255, G312, G318, G319, G931, G934, R470, G10, G11, G12, G13, G20, G21, G22, G32, G35, G36, G37, G40, G41, R56 |
| ***Comorbidities*** | |
| Cardiac | 4275, 4271, 42741, 4233, 41000, 41001, 41010, 41011, 41020, 41021, 41030, 41031, 41040, 41041, 41050, 41051, 41060, 41061, 41070, 41071, 41080, 41081, 41090, 41091, I462, I468, I469, I472, I4901, I314, I21 |
| Respiratory | 4800, 4801, 4802, 4803, 4808, 4809, 4820, 4821, 4822, 4829, 4830, 4831, 4838, 48230, 48231, 48232, 48239, 48240, 48241, 48242, 48249, 48281, 48282, 48283, 48284, 48289, 481, 485, 486, 99731, 99732, 5121, 5185, 51882, 51881, 51851, 51853, 51884, 9672, 5100, J14, J13, J181, J120, J121, J122, J129, J150, J151, J153, J154, J150, J151, J153, J154, J158, J155, J156, A481, J158, J159, J157, J160, J168, J180, J189, J1281, J1289, J1520, J1529, J15211, J15212, J95851, J9589, J95811, J80, R0603, J9600, J9690, J9620, J95821, J95822, 5A1955Z |
| Renal | 5854, 5855, 5856, V4511, 40301, 40311, 40391, 40402, 40403, 40412, 40413, 40492, 40493, 5856, 586, N184, N185, N186, Z992, N19, I120, N186, I1311 |
| Need for blood transfusion | 990, 30233H0, 30233N0, 30243H0, 30243N0, 30253H0, 30253N0, 30263H0, 30263N0, 30233H1, 30243H1, 30253H1, 30263H1, 30233H0, 30233N0, 30233W0, 30243H0, 30243N0, 30243W0, 30253H0, 30253N0, 30253W0, 30233H0, 30233N0, 30233W0, 30243H0, 30263H0, 30263N0, 30263W0, 30233H1, 30243H1, 30253H1, 30263H1, 30233N1, 30233P1, 30243N1, 30243P1, 30253N1, 30253P1, 30263N1, 30263P1, 30233R1, 30243R1, 30253R1, 30263R1, 30233T1, 30233V1, 30233W1, 30243T1, 30243V1, 30243W1, 30253T1, 30253V1, 30253W1, 30263T1, 30263V1, 30263W1, 30233J1, 30233K1, 30233L1, 30233M1, 30243J1, 30243K1, 30243L1, 30243M1, 30253J1, 30253K1, 30253L1, 30253M1, 30263J1, 30263K1, 30263L1, 30263M1, 3E033GC, 3E043GC, 3E053GC, 3E063GC, 30233Q1, 30243Q1, 30253Q1, 30263Q1 |
| Infectious | 038, 99591, 99592, 9993, A419, 99851, 99859, 99831, 99832, 9985, A409, A412, A4101, A4102, A411, A403, A414, A4150, A413, A4151, A4152, A4153, A4159, A4189, A419, R6520, T80219A, T80211A, T80212A, T8022XA, T8029XA, T814XXA, K6811, T8132XA, T8131XA, T814XXA, K6811 |
| Stroke | 431, 4320, 4321, 4329, 430, 43301, 43311, 43321, 43331, 43381, 43391, 43401, 43411, 43491, 4370, 4371, 4374, 4375, 4377, 4379, 99701, 99702, I619, I621, I620, I619, I629, I609, I6322, I63139, I63239, I63019, I63119, I63219, I6359, I6320, I6330, I6340, I6350, I672, I6781, I6782, I6789, I677, I675, G454, I679, G9781, G9782, I97811, I97821 |
| Thrombotic | 4511, 4512, 45181, 4519, 4532, 45340, 45341, 45342, 4538, 4539, 4151, 41511, 41512, 41519, I8010, I80209, I803, I80219, I809, I82220, I82409, I82419, I82429, I82439, I824Y9, I82449, I82499, I824Z9, I82619, I82629, I82609, I82A19, I82B19, I82C19, I82290, I82890, I8291, T800XXA, T81718A, T8172XA, T82817A, T82818A, I2690, I2699 |
